# Long-term outcome of combined radiologic and surgical strategy for the management of biliary complications after pediatric liver transplantation

**DOI:** 10.1186/s13104-024-06735-6

**Published:** 2024-03-20

**Authors:** Ana M. Calinescu, Sébastien Monluc, Stephanie Franchi-Abella, Dalila Habes, Gabrielle Weber, Marion F. Almes, Jerome Waguet, Emmanuel Jacquemin, Virginie Fouquet, Jordi Miatello, Geraldine Hery, Catherine Baujard, Emmanuel Gonzales, Sophie Branchereau, Florent Guérin

**Affiliations:** 1 Paediatric Surgery Unit, Université Paris-Saclay, Assistance Publique-Hôpitaux de Paris, Bicêtre Hospital, 78 Rue du Général Leclerc, 94270 Le Kremlin-Bicêtre, France; 2 University Center of Pediatric Surgery of Western Switzerland, Geneva University Hospitals, Division of Pediatric Surgery, University of Geneva, 6 Rue Willy Donze, 1205 Geneva, Switzerland; 3 Assistance Publique-Hôpitaux de Paris, Bicêtre Hospital, Epidemiology and Public Health Department, Université Paris-Saclay, Le Kremlin-Bicêtre, France; 4 Assistance Publique-Hôpitaux de Paris, Bicêtre Hospital, Pediatric Radiology Unit, Université Paris-Saclay, Le Kremlin-Bicêtre, France; 5 Assistance Publique-Hôpitaux de Paris, Bicêtre Hospital, Pediatric Hepatology and Pediatric Liver Transplantation Unit, Université Paris-Saclay, Le Kremlin-Bicêtre, France; 6 Hépatinov, Inserm U 1193, National Reference Centre for Rare Pediatric Liver Diseases, FSMR FILFOIE, ERN RARE LIVER, Assistance Publique-Hôpitaux de Paris, Bicêtre Hospital, Pediatric Hepatology and Pediatric Liver Transplantation Unit, Université Paris-Saclay, Le Kremlin-Bicêtre, France; 7 Assistance Publique-Hôpitaux de Paris, Bicêtre Hospital, Department of Pediatric and Neonatal Intensive Care, Université Paris-Saclay, Le Kremlin-Bicêtre, France; 8 Assistance Publique-Hôpitaux de Paris, Bicêtre Hospital, Anesthesia Department, Université Paris-Saclay, Le Kremlin-Bicêtre, France

**Keywords:** Biliary complications, Pediatric liver transplantation, Repeated radiologic procedures

## Abstract

**Objectives:**

We aimed to analyze the risk factors for management failure of BC after pediatric liver transplantation (pLT) by retrospectively analyzing primary pLT performed between 1997 and 2018 (n = 620 patients).

**Results:**

In all, 117/620 patients (19%) developed BC. The median (range) follow-up was 9 (1.4–21) years. Patient survival at 1, 5 and 10 years was 88.9%, 85.7%, 84.4% and liver graft survival was 82.4%, 77.4%, and 74.3% respectively. Graft not patient survival was impaired by BC (p = 0.01). Multivariate analysis identified the number of dilatation courses > 2 (p = 0.008), prolonged cold ischemia time (p = 0.004), anastomosed multiple biliary ducts (p = 0.019) and hepatic artery thrombosis (p = 0.01) as factors associated with impaired graft survival. The number of dilatation courses > 2 (p < 0.001) and intrahepatic vs anastomotic stricture (p = 0.014) were associated with management failure. Thus, repeated (> 2) radiologic dilatation courses are associated with impaired graft survival and management failure. Overall, graft but not patient survival was impaired by BC.

**Supplementary Information:**

The online version contains supplementary material available at 10.1186/s13104-024-06735-6.

## Introduction

Biliary complications (BC) are a common occurrence after pediatric liver transplantation (pLT), accounting for 10–35% of cases [1–18]. While interventions such as percutaneous transhepatic cholangiography with balloon cholangioplasty (PTC-C), endoscopic retrograde cholangiopancreatography (ERCP) and surgery can be used to manage these complications, PTC-C is considered the gold standard treatment for biliary strictures in pLT [19]. Surgical revision is now reserved as a last resort for these patients [18, 20, 21].

However, managing biliary strictures and leaks in pLT can be challenging, requiring repeated radiologic procedures, long-term biliary drainage and even repeated surgeries [22, 23] with a significant impact on graft, patient survival and quality of life [6, 9, 24]. The outcomes of repeated PTC-C in pLT  are inconsistent, complicating the determination of factors associated with treatment failure and the establishment of long-term outcomes [18].

Therefore, the primary aim was to identify factors leading to the failure to manage these complications. The secondary aim was to define long-term outcomes of BC.

## Main text

### Methods

#### Patients

All primary pLT procedures performed at our center between 1997 and 2018 with a minimal follow-up of 1 year were retrospectively reviewed. Exclusion criteria were: previous pLT and multiple organ transplantation to exclude BC related to prolonged cold ischemia times. Bile leaks that did not have a percutaneous cholangiography or surgery and were managed conservatively were excluded. All BC identified by the need for either percutaneous cholangiography or surgery were included. Patient characteristics including age at pLT, gender, indication for pLT, type of donor, type of liver graft, cold ischemia time, number of bile ducts within the anastomosis, type of biliary anastomosis, hepatic artery thrombosis/stenosis and episodes of biopsy-proven acute rejection were recorded. Initial immunosuppression was based on cyclosporine or tacrolimus and steroids from 1997 to 2003 and on basiliximab plus tacrolimus since 2003. Of note, split and reduced liver grafts were all performed ex-situ. Details concerning the surgical procedure of biliary anastomosis, diagnostic criteria, treatment of BC and outcome are detailed in Additional file 1.

#### Statistical analysis

SAS version 9.4 (SAS Institute, Cary, NC) was used for statistical analysis. Data were quoted as median (range). Comparisons were performed. Categorical variables were compared with the Chi-square test or a Fisher exact test. Continuous data were compared with a Mann–Whitney test. Survival curves and confidence intervals were estimated with the Kaplan–Meier method. To evaluate the hazard ratios for the number of courses of PTC-C, the extended Cox model for time-dependent variables was used. Hazard ratios were estimated with univariate Cox proportional hazards models. Wald test was performed to determine the variables in univariate models with a p-value of < 0.20. These variables were then used to construct the multivariate model, with backward elimination. All the results were considered significant at a p-value of < 0.05.

### Results

#### Patient characteristics

In the 620 pLT recipients, 117 (19%) developed a BC including 16 bile leaks (strictures were identified in all 16 patients). One hundred six patients had a primary PTC-C and 11 primary surgery.

The demographic data and clinical characteristics of the 117 patients with BC are summarized in Table 1. Median follow-up was 9 years (range 1.4—21 years) (Table 1).

**Table 1:** Characteristics of the patients with biliary complications, LT: Liver transplantation

Baseline characteristics N = 117	%	Nr.
**Age at LT**		
21 (5–135) months		
**Patient sex**		
Male	42	49
Female	58	68
**Indications for LT**		
Biliary atresia	68	79
Fulminant hepatic failure	10	12
Other	22	26
**Type of donor**		
Living related	28	33
Deceased	72	84
**Type of liver graft**		
Left lateral segment deceased	48	56
Left lateral segment living	28	33
Left lobe	13	15
Whole liver	5	6
Other	6	7
**Cold ischemia time (min)**		
≤ 600	64	75
> 600	28	33
Unknown	8	9
**Number of bile ducts anastomosis**		
1	69	81
≥ 2	16	19
Unknown	5	6
**Biliary anastomosis type**		
Bilioenteric	96	112
Bilio-biliary	4	5
**Hepatic artery thrombosis/stenosis**	22	26
**Acute rejection**	38	45

#### Management of BC

**Fig. 1:**
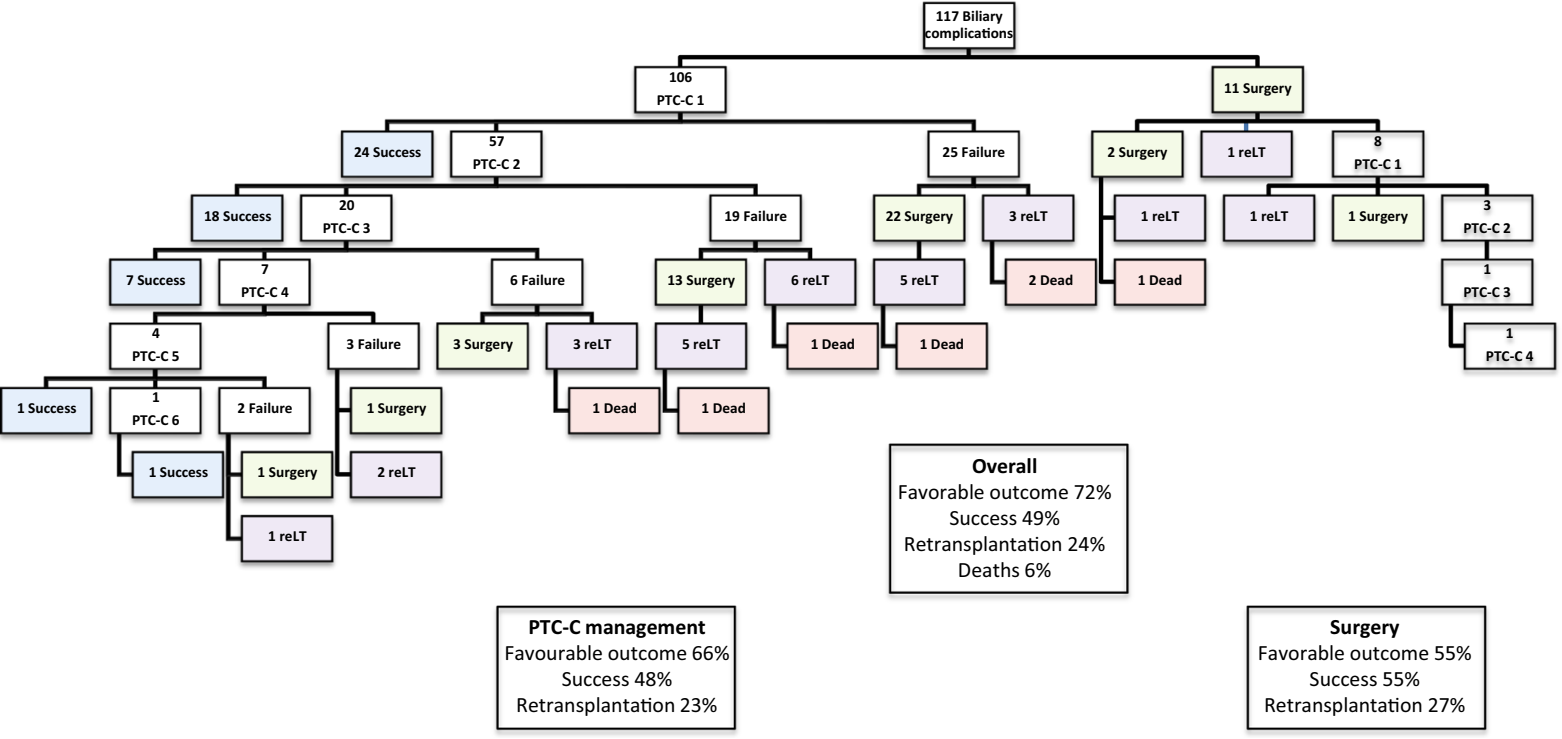
Flowchart of patients with biliary complications after pediatric liver transplantation. *PTC-C* percutaneous transhepatic cholangiography with balloon cholangioplasty, *Re LT* redo-liver transplantation

Fifty-seven patients 57 (49%) achieved success with either percutaneous transhepatic cholangiography with balloon cholangioplasty (PTC-C) (51 patients) or primary surgery (6 patients). Additionally, 25 patients (23%) had favorable outcomes, without the need for subsequent surgery or pLT. On the other hand, 28 patients (24%) required retransplantation, and there were 7 deaths (6%), 6 of which occurred after the retransplantation procedure (Fig. 1).

Within the primary PTC-C group, at the end of the study, 51 patients (48%) were successfully treated only with PTC-C, including 4 out of 5 patients (80%) with duct-to-duct anastomosis. The 5th patient needed an ERCP after one failed PTC-C with a favorable outcome afterwards. A secondary surgery was necessary for 40 patients (38%), while 25 patients (21%) required a second LT. The median number of PTC-C courses was 2 (range 1–6), and the median dilatation sessions per PTC-C course was 2 (range 1–7). The recurrence rate was 54% (57/106 patients) after the 1st PTC-C, compared to 35% after the 2nd (20/55 patients) and 3rd (7/20 patients) PTC-C course (p = 0.010). Failure rates were 23% (25/106 patients), 33% (19/57 patients), 30% (6/20 patients), 42% (3/7 patients), and 50% (2/4 patients) after the 1st, 2nd, 3rd, 4th, and 5th PTC-C course, respectively. The time of drainage for a PTC-C course was less than 3 weeks for 73% (74/106 patients) during the 1st treatment course, 70% (47/71 patients) during the 2nd treatment course, and 84% (26/31 patients) for the 3rd treatment course (Additional file 2). Procedure-related complications, rates of haemobilia, sepsis and cholangitis are displayed in Additional file 3.

Fifty-one patients (44%) underwent surgical procedures for their BC: 11 (21%) had primary surgery, and 40 (78%) had secondary surgery with a median of 2 previous PTC-C courses (range 1–5). The retransplantation rate of 23% (24/106) was not different between primary PTC-C and the primary surgery group 27% (3/11) (p = 0.796), and was not different between the primary PTC-C group without consecutive surgery 23% (15/66) when compared to the surgery group 23.5% (12/51) (p = 0.949).

#### Risk factors for management failure or graft loss

In the primary PTC-C group, factors associated with *management failure* were, in univariate analysis: hepatic artery thrombosis, the intrahepatic vs. anastomotic stricture, lithiasis at first PTC-C, and the number of PTC-C courses (Table 2). In multivariate analysis, failure of PTC-C was associated with the number of PTC-C courses (p < 0.001) and intrahepatic vs anastomotic stricture (p = 0.014) (Table 2).

**Table 2:** Univariate and multivariate analysis of risk factors for primary percutaneous transhepatic cholangiography with balloon cholangioplasty (PTC-C) group treatment failure. LT: Liver transplantation

Variables	Hazard ratio [CI 95%]	p
**Number of PTC-C**^a^		** < 0.001**
1	**1**	
2	**7.296 [3.180–16.739]**	
≥ 3	**15.509 [5.185–46.396]**	
Female sex	1.763 [0.992**–**3.133]	0.053
Liver disease		0.720
Biliary atresia	1	
Fulminant hepatitis	0.691 [0.272–1.758]	
Other	0.884 [0.450–1.734]	
Type of LT		0.630
Left lateral segment (organ donation)	1	
Left lobe	1.226 [0.542–2.771]	
Left lateral segment (living donation)	1.517 [0.799–2.878]	
Whole liver	1.488 [0.442–5.010]	
Cold ischemia time > 600 min	1.184 [0.664–2.110]	0.570
Number of bile ducts anastomosis = 2	1.283 [0.656–2.511]	0.470
Bilio-biliary anastomosis	0.386 [0.053–2.795]	0.350
Number of arteries = 2	1.689 [0.524–5.447]	0.380
**Hepatic artery status at 1 month**		**0.014**
No arterial issues	**1**	
Thrombosis	**2.292 [1.261–4.166]**	
Stenosis	**0.426 [0.058–3.122]**	
Acute rejection	0.812 [0.465–1.416]	0.460
Cholangitis at first PTC-C	1.410 [0.799–2.488]	0.240
Cytolysis at first PTC-C	1.074 [0.562–2.055]	0.830
Cholestasis at first PTC-C	1.729 [0.622–4.807]	0.290
Age at first PTC-C^b^	1.001 [0.919–1.089]	0.990
Time between LT and first PTC-C^b^	1.000 [0.987–1.013]	0.990
**Intrahepatic stenosis at first PTC-C**	**1.873 [1.088–3.222]**	**0.023**
Extrahepatic stenosis at first PTC-C	0.876 [0.349–2.200]	0.770
**Anastomotic stenosis at first PTC-C**	**0.454 [0.257**–**0.801]**	**0.006**
**Lithiasis at first PTC-C**	**2.352 [1.092**–**5.067]**	**0.028**
Drain type after first PTC-C		0.260
External drain	1	
External drain + internal–external drain	2.071 [0.871–4.923]	
Internal–external drain	1.165 [0.458–2.964]	
Duration of the first drain^b^	1.191 [0.813–1.746]	0.370
Complications after first PTC-C		0.270
No complication	1	
Minimal hemobilia	1.660 [0.653–4.220]	
Important hemobilia	0.755 [0.182–3.129]	
Cholangitis	0.358 [0.110–1.160]	
Sepsis	0.612 [0.189–1.984]	
**Multivariate analysis**		
PTC-C Nr. 2 (ref.1)	**6.764** [2.957–15.571]	** < .0001**
PTC-C Nr. 3 or more (ref.1)	**15.511** [5.157–46.650]	** < .0001**
Anastomotic stenosis 1 (ref none)	**0.489** [0.276–0.865]	**0.014**

Bold was used for statistically significant values

^a^Analyzed as a time-dependent variable

^b^Continuous variables

In the primary PTC-C group, factors associated with *graft failure or patient death* were, in univariate analysis: prolonged cold ischemia time, hepatic artery thrombosis, lithiasis at first PTC-C, duration of the first biliary drainage longer than 3 weeks, and the number of PTC-C courses (Additional file 4). In multivariate analysis, prolonged cold ischemia time (p = 0.004), hepatic artery thrombosis (p = 0.010), the number of PTC-C courses (p = 0.008), and two-bile ducts anastomosis (p = 0.019) were associated with graft loss or patient death (Additional file 4). The drain duration of more or less than 3 weeks was not associated to PTC-C treatment failure in the multivariate analysis (p = 0.370) (Additional file 4).

Finally, the study found that the management failure rates in the primary PTC-C group after the 4th and 5th PTC-C course were 42% and 50%, respectively, which were higher than the overall 28% failure rate of the surgical management group, although the difference was not statistically significant (p = 0.435 and p = 0.399 respectively).

#### Long-term survival after BC management

In our pLT cohort, the overall survival (OS) rates at 1, 5, and 10 years were 82.4%, 77.4%, and 74.3%, respectively. Patients *without* BC had OS rates of 86.7%, 83.5%, and 82.2% at 1, 5, and 10 years, respectively, and graft survival (GS) rates of 79.5%, 75.9%, and 73.2%, respectively (Additional file 5). Patients *with* BC had OS rates of 98.3%, 95.2%, and 94% at 1, 5, and 10 years, respectively, and GS rates of 94.9%, 83.8%, and 78.9%, respectively (Additional file 5). However, when using the extended Cox model for time-dependent variables to manage the immortal time bias and compare patients with and without BC, patients with BC had a shorter GS than patients without BC [HR = 2.483(1.568–3.934), p < 0.001]. The same Cox model did not show impaired patient OS for patients *with* BC compared to patients *without* BC [HR = 0.948(0.449–2.002)].

In the primary PTC-C group, GS was impaired [HR = 2.4(1.5–3.9), p = 0.010], as well as within primary surgery group (p = 0.02).

### Discussion

BC are among the most frequent complications after pLT [3–5, 9, 12, 13, 25], their treatment consisting mainly of PTC-C. To our knowledge, this is the first study to report an association between PTC-C failure rates and repeated radiologic procedures. For graft failure, besides repeated radiologic procedures, long drainage periods were also a risk factor.

The high failure rates of PTC-C with repeated radiologic procedures (i.e. > 2) found in our series might be explained by fibrosis with retraction and scarred biliary tissue following repeated radiologic instrumentations of the bile ducts, etiologic findings already published by others [26, 27]. Even if centers report evaluating a surgical revision after 1–2 failed PTC-C for BC occurring 12 months after pLT [18], our findings provide evidence-based support for the clinician’s decision. Because failure rates increase significantly after 3rd dilatation course, it appears that those strictures are unlikely to respond to subsequent dilatation attempts.

Long drainage periods after dilatation for biliary strictures in pediatric LT seem to be associated with high success rates in the available literature (Additional file 6) but success rates in the treatment of BC are difficult to compare [28]: frequently definitions are lacking; when described, the success reported might be technical, clinical or associated with improved patency rates of the biliary anastomosis [22, 29, 30]. The difference between our study and the existing literature could be attributed to the different management strategies: short drainage period and external drainage in our center versus longer drainage period and internal–external drainage in the other series [18]. The Society of Pediatric Liver Transplantation analysis failed to show the optimal management type (PTC-C, ERCP) or timing between PTC-C procedures (more or less than 3 weeks) underlining the need for a prospective study [17]. The success of short draining periods that are used in our experience supports the concept of biodegradable stents for the treatment of biliary strictures in pLT [31].

The prolonged graft ischemia time, hepatic artery thrombosis, the number of duct anastomoses, and the number of PTC-C courses were also significant, underlying the intrication of graft survival with the risk of repeated and probably unsuccessful percutaneous procedures in small bile duct structures [26, 27, 32]. Several other studies investigating the risk factors for BC found donor, immunological and ischemic factors [3, 11, 17, 33]. Nevertheless, their impact on treatment failure was not further investigated in our series. The high hepatic artery thrombosis rates found are explained by the long period of the study starting in 1997. Furthermore, the thrombosis rate encompasses both intra and postoperative occurrences with a significant resolution observed within the first month, yielding an 8% thrombosis rate at one month. Multiple biliary anastomoses are known to increase the risk of BC in pLT [6, 34]; furthermore, in our series, the multivariate analysis identified multiple bile ducts anastomosis to be associated with increased risk of graft failure after PTC-C management of BC. A trend without statistical significance was already reported [34, 35]. While the diameter of the bile duct used for reconstruction is indeed a critical factor in biliary anastomosis, the type of graft was not associated in our series with PTC-C treatment failure or graft failure.

The ratio of patients having either successful or favourable outcomes at the end of follow-up was as high as 72% with 48% having PTC-C successfully dilated strictures and free of recurrent strictures throughout extended follow-up. Throughout the literature, there are different PTC-C protocols (Additional file 6) and results vary accordingly. The length of the PTC-C course used within our series is shorter than the average of the above-mentioned series, approaching the model of the “three-session protocol” of Oggero et al. [36]. The authors report a 71.4% success rate after the first PTC-C [36]. The rationale for this short period [7–10 days] between dilatations is based on the hypothesis of a first dilatation injury followed by a repair process occurring in the 45 days after tissue injury (i.e. first dilatation session) with a peak deposit of fibroblasts at day 30 [37]. By keeping the drainage periods short, we hypothesized a reduced drainage-induced inflammation and thus a shortened healing time.

The place of surgical treatment in the management of BC after pediatric LT is not well defined: in the past, it was the only treatment available for BC [3, 38], being progressively indicated only in the management of bile leaks and in case of failure of interventional radiology [35, 39, 40], even if surgery is still being used as first-line therapy in some centers [21]. Surgical revision was successful in our series for saving strictures not amenable to radiologic treatment. Even if not statistically significant, failure rates after the 3rd and 4th PTC-C were higher than after the surgical treatment, pointing to the need to evaluate the interest for earlier evaluation for surgical management after the 2nd PTC-C.

In our series, 66% of patients managed surgically for BC required further intervention, either PTC-C or additional surgery. This is in contrast to another series where only 20% needed repeat surgery, with a retransplantation rate of 12% [21]. The discrepancy may stem from the latter series exclusively using surgery for all biliary complications, tracked over a variable follow-up period averaging 92 months [21]. While a second surgery could reduce hospital stays and be less painful than multiple PTC-Cs, the benefits and drawbacks of each approach warrant further comparative research to establish optimal management strategies.

## Limitations

This retrospective series offers an overview of the long-term outcomes of BC over 21 years. While management bundles changed little during the study period, medical tools and fine-tuning of clinical practices may have impacted the outcomes.

Patient age, and thus weight and size, vary from neonates to nearly teenagers.

Furthermore, the study was performed over a long study period. Some variables for data that could have been of interest (i.e. donor data, immunological data) were not available in the revised records.

**To conclude**, in the long-term, graft and not patient survival is impaired for patients with BC after pediatric LT. Repeated (i.e. > 2 PTC-C courses) radiologic procedures are associated with impaired graft survival and management failure. Thus, an alternative surgical treatment could be proposed beyond two dilatation courses in the management of BC after pediatric liver transplantation.

### Supplementary Information

**Additional file 1. **Methods: the surgical procedure of biliary anastomosis, diagnostic criteria, treatment of biliary complications,outcome.

**Additional file 2. **Drainage duration for each percutaneous transhepatic cholangiography with balloon cholangioplasty (PTC-C) course of treatment for biliary complications after pediatric liver transplantation

**Additional file 3. **Society of interventional radiology (SIR) classification of complications of percutaneous transhepatic cholangiography with balloon cholangioplasty (PTC-C) for biliary complications after pediatric liver transplantation (N=106)

**Additional file 4. **Uni and multivariate analysis of risk factors for graft failure in the primary percutaneous transhepatic cholangiography with balloon cholangioplasty (PTC-C) group. PTC-C: refers to a PTC-C treatment course.

**Additional file 5. **Patient and liver graft survival within the pediatric liver transplantation cohort and biliary complications cohort.

**Additional file 6. **Treatment protocol for percutaneous transhepatic balloon cholangioplasty in pediatric liver transplantation and success rates. ERCP: endoscopic retrograde cholangiopancreatography, LT: liver transplantation, n.a.: not applicable.

## Data Availability

The datasets used and/or analysed during the current study are available from the corresponding author upon reasonable request.

## Abbreviations

pLT: Pediatric liver transplantation
BC: Biliary complications
PTC-C: Percutaneous transhepatic cholangiography with balloon cholangioplasty
ERCP: Endoscopic retrograde cholangiopancreatography
OS: Overall survival
GS: Graft survival
