# Supplementary material for: Long-term outcome of combined radiologic and surgical strategy for the management of biliary complications after pediatric liver transplantation
Source: BMC Res Notes. 2024 Mar 20;17:86. doi: 10.1186/s13104-024-06735-6 (PMC10953252; doi:10.1186/s13104-024-06735-6)
Supplement: Supplementary file 1 — Additional file 1. Methods: the surgical procedure of biliary anastomosis, diagnostic criteria, treatment of biliary complications,outcome. [file 13104_2024_6735_MOESM1_ESM.docx]

**Additional material 1**

- 1. *The surgical procedure of biliary anastomosis*

In patients with biliary atresia or preexisting bile duct conditions, a hepaticojejunostomy

with Roux-en-Y loop was used. The duct-to-duct anastomosis was performed if anatomically feasible. The hepaticojejunostomy and duct-to-duct reconstruction were performed using monofilament absorbable sutures in an interrupted or continuous suture, at the discretion of the operating surgeon, employing microsurgery principles. No T-tubes or stents were used. In the case of multiple bile ducts, the anastomosis was performed conjoinedly or separately according to the distance between ducts and the possibility of performing a bile duct plasty.

- 1. *Diagnostic criteria*

Biliary strictures were suspected based on clinical findings (jaundice, pruritus, cholangitis), abnormal liver tests, gamma-glutamyl transferase (GGT) and/or bilirubin abnormal values and non-invasive imaging studies (ultrasound and/or computed tomography (CT) and/or magnetic resonance imaging (MRI)). Diagnosis of biliary stricture was confirmed by percutaneous transhepatic cholangiography (PTC).

Bile leak was diagnosed if bile-like ascites is drained and an elevated bilirubin concentration in the drainage fluid is found (three times more elevated than the bilirubin serum values).

Biloma was defined as a perihepatic fluid collection with a typical appearance on imaging (ultrasound and/or CT and /or MRI) and found to be of biliary origin after percutaneous drainage.

- 1. *Treatment of BC*

Radiologic and surgical treatment options are detailed below. Of note, endoscopic management is also available in our center and was considered for BC of duct-to-duct anastomosis.

2.4.1 Radiologic treatment of BC

All patients with a suspicion of biliary stricture and bile duct dilatation had a PTC. Opacification of the biliary tree was performed under sonographic guidance through primary or secondary biliary radicals. After demonstrating the stricture(s), a guidewire was passed through the stenosis and balloon cholangioplasty was performed. As per local protocol, an external drainage catheter 6 to 8 Fr according to the patient’s weight was left above the stricture. One week to 10 days later, a further PTC and cholangioplasty (PTC-C) if necessary were performed according to the degree of stricture. External biliary drainage was removed 48-72 hours after a “clamping test”. Liver biochemistry and a liver ultrasound were performed 3 days following clamping. The test was considered successful (i.e. biliary catheter can be safely removed) if the patient did not experience fever, pruritus, or worsening of the liver biochemistry. For a *PTC-C* *course*, the minimum length of stay was 10-12 days comprising usually one or more *PTC-C* *sessions* (if needed). The number of PTC-C sessions per course was determined by the results of the cholangiogram at the beginning of the next procedure. Thus, a *PTC-C* *session* was defined as the *dilatation procedure itself*; the *PTC-C* *course* was defined as *all dilatation sessions* needed to conclude either for a good result or a failure, defined as below (i.e. Outcome subchapter). Recurrence, defined as biliary stricture reappearing after a PTC-C course, was treated primarily with radiologic treatment. The number of PTC-C courses (each may comprise several dilatation sessions) was taken as a variable of interest. Exceptionally, internal-external drains were used.

Bile leaks causing bilomas were treated with percutaneous drainage with or without PTC.

Complications after PTC-C were graded according to the Society of Interventional Radiology (SIR) classification^25^. Minor complications were stratified in A: no therapy, no consequence and B: nominal therapy, no consequence; including overnight admission for observation only. Major complications were stratified in C: requires therapy, minor hospitalization (<48h), D: requires major therapy, unplanned increase in the level of care, prolonged hospitalization (>48h), E: permanent adverse sequelae, F: death^25^.

The variables taken into consideration were: the number of PTC-C dilatation sessions per course and the overall number of PTC-C courses, gender, disease necessitating LT, type of graft, cold ischemia time, number of bile duct anastomosis, type of biliary anastomosis, number of arterial anastomoses, hepatic artery thrombosis/stenosis, acute rejection, cholangitis at first PTC-C course, increased hepatic enzymes (alanine aminotransferase (ALT) or aspartate aminotransferase (AST)) at first PTC-C course, increased GGT and/or bilirubin at first PTC-C course, age at first PTC-C course, the time between LT and first PTC-C course, intrahepatic stenosis at first PTC-C course, extrahepatic stenosis at first PTC-C course, anastomotic stenosis at first PTC-C course, lithiasis at first PTC-C course, internal or external drain after the first PTC-C course, duration of the biliary drainage catheter at the first PTC-C course, complications after the first PTC-C course. Cholestasis was defined as abnormal GGT and/or bilirubin. Minimal haemobilia was defined as self-resolving haemobilia not needing any further therapy nor hospital stay longer than 48h (i.e. blood transfusions). Important haemobilia was defined as haemobilia needing further therapy (i.e. blood transfusions) and longer hospitalization than 48h.

- - 1. Surgical treatment of BC

For strictures, surgical intervention was reserved for cases where radiologic treatments had failed. The decision to proceed with surgical revision was made on a case-by-case basis by a multi-disciplinary team consisting of transplant surgeons, pediatric hepatologists, and interventional radiologists. For very localized anastomotic strictures that had failed radiologic procedures, a widening plasty was suggested. This involved opening the anterior layer of the hepaticojejunostomy, cutting the fibrosis on the anterior part, and redoing the anterior bilio enteric suture. In cases where stricture affected only the biliary drainage of one segment, a bilio enteric anastomosis was performed using the existing Roux-en-Y limb and the related segment. Long anastomotic strictures were treated with a redo hepaticojejunostomy using the already existing Roux-en-Y limb. Indwelling biliary drainage catheters were preferred to guide the location of the bile ducts within the cut surface of the liver graft. Relapses after surgical treatment were primarily treated with radiologic treatment. If radiological and/or surgical treatment failed and cholangiopathy progressed, retransplantation was the next option.

In case of bile leaks, major persistent, or early (less than 3 months after LT) bile leaks were treated with surgical intervention, with a segmental or complete redo of the hepaticojejunostomy.

Complications after surgery were graded according to the Clavien-Dindo system^26^.

- 1. *Outcome*

A favourable outcome for both PTC-C and surgical treatment was defined as no need for another surgical procedure or retransplantation. Failure of PTC-C management was defined as either failure of cholangiographic resolution of the stricture without further radiological treatment possible or the need for either surgery or retransplantation. Failure of surgical management was defined as either relapse of the stricture without further radiologic treatment or the need for surgery or retransplantation. Patients having either two surgeries or PTC-C plus surgery have by definition failure of surgery or PTC-C but they can have a favourable outcome if they don’t require either a further biliary surgical procedure or a second transplantation at the end of procedures. Either graft loss or patient death was considered both an unfavourable outcome and treatment failure too.
