# Supplementary material for: Long-term outcome of combined radiologic and surgical strategy for the management of biliary complications after pediatric liver transplantation
Source: BMC Res Notes. 2024 Mar 20;17:86. doi: 10.1186/s13104-024-06735-6 (PMC10953252; doi:10.1186/s13104-024-06735-6)
Supplement: Supplementary file 2 — Additional file 2. Drainage duration for each percutaneous transhepatic cholangiography with balloon cholangioplasty (PTC-C) course of treatment for biliary complications after pediatric liver transplantation [file 13104_2024_6735_MOESM2_ESM.docx]

**Additional material** **2** Drainage duration for each percutaneous transhepatic cholangiography with balloon cholangioplasty (PTC-C) course of treatment for biliary complications after pediatric liver transplantation

| **Drain duration** | **1^st^ PTC-C**  **N=106** | | **2^nd^ PTC-C**  **N=71** | | **3^rd^ PTC-C**  **N=31** | |
| --- | --- | --- | --- | --- | --- | --- |
| < 3 weeks | 74 | (73%) | 47 | (70%) | 26 | (84%) |
| 3 weeks – 3 months | 17 | (17%) | 17 | (25%) | 4 | (13%) |
| >3 months | 10 | (10%) | 3 | (5%) | 1 | (3%) |
