# Supplementary material for: Long-term outcome of combined radiologic and surgical strategy for the management of biliary complications after pediatric liver transplantation
Source: BMC Res Notes. 2024 Mar 20;17:86. doi: 10.1186/s13104-024-06735-6 (PMC10953252; doi:10.1186/s13104-024-06735-6)
Supplement: Supplementary file 3 — Additional file 3. Society of interventional radiology (SIR) classification of complications of percutaneous transhepatic cholangiography with balloon cholangioplasty (PTC-C) for biliary complications after pediatric liver transplantation (N=106) [file 13104_2024_6735_MOESM3_ESM.docx]

**Additional material 3**

Society of interventional radiology (SIR) classification of complications of percutaneous transhepatic cholangiography with balloon cholangioplasty (PTC-C) for biliary complications after pediatric liver transplantation (N=106)

| **SIR** | **Type** | **1^st^ PTC-C**  **N=106** | | **2^nd^ PTC-C**  **N=71** | | **3^rd^ PTC-C**  **N=31** | |
| --- | --- | --- | --- | --- | --- | --- | --- |
| B | Minimal hemobilia | 6 | (6%) | 8 | (12%) | 6 | (20%) |
| C | Major hemobilia | 4 | (4%) | 1 | (2%) |  | |
| D | Cholangitis | 15 | (15%) | 7 | (11%) | 4 | (13%) |
|  | Sepsis | 7 | (7%) | 1 | (2%) |  | |
