# Supplementary material for: Long-term outcome of combined radiologic and surgical strategy for the management of biliary complications after pediatric liver transplantation
Source: BMC Res Notes. 2024 Mar 20;17:86. doi: 10.1186/s13104-024-06735-6 (PMC10953252; doi:10.1186/s13104-024-06735-6)
Supplement: Supplementary file 4 — Additional file 4. Uni and multivariate analysis of risk factors for graft failure in the primary percutaneous transhepatic cholangiography with balloon cholangioplasty (PTC-C) group. PTC-C: refers to a PTC-C treatment course. [file 13104_2024_6735_MOESM4_ESM.docx]

**Additional material 4**

Uni and multivariate analysis of risk factors for graft failure in the primary percutaneous transhepatic cholangiography with balloon cholangioplasty (PTC-C) group. PTC-C: refers to a PTC-C treatment course.

|  | **Univariate Cox Model** | | **Multivariate Cox Model** | |
| --- | --- | --- | --- | --- |
|  | **Hazard ratio [CI 95%]** | **p** | **Hazard ratio [CI 95%]** | **p** |
| **Number of PTC-C**  **(ref 1)** † |  | **0.002** |  | **0.008** |
| **1**  **2**  **3** | **1**  **6.684 [1.846 - 24.197]**  **11.800[2.964 -46.977]** |  | **1**  **8.389 [1.452 - 48.464]**  **19.602 [2.946 - 130.444]** |  |
| Liver disease (ref Biliary atresia) |  | 0.114 |  | 0.154 |
| Biliary atresia  Fulminant hepatitis  Other | 1  2.849 [1.027 - 7.905]  0.975 [0.320 - 2.970] |  | 1  3.478 [0.665 - 18.188]  0.463 [0.100 - 2.149] |  |
| **Cold ischemia time (ref <600)** | **2.469 [1.069 - 5.705]** | **0.034** | **6.731 [1.831 - 24.741]** | **0.004** |
| **Number of bile duct anastomosis (ref 1)** | **1.981 [0.799 - 4.911]** | **0.139** | **4.508 [1.268 - 16.018]** | **0.019** |
| **Hepatic artery status at 1 month (ref normal)** |  | **0.001** |  | **0.010** |
| **Normal**  **Thrombosis**  **Stenosis** | **1**  **5.173 [2.316 - 11.555]**  **1.527 [0.198 - 11.791]** |  | **1**  **5.139 [1.485 - 17.785]**  **0.331 [0.032 - 3.416]** |  |
| Intrahepatic stenosis at 1^st^ PTC-C (ref absence) | 2.068 [0.928 - 4.609] | 0.075 | 0.295 [0.084 - 1.031] | 0.055 |
| Duration of the first drain | 1.719 [1.013 - 2.919] | 0.044 | 2.013 [0.822 - 4.930] | 0.125 |

†:  Analyzed as a time-dependent variable
