# Supplementary material for: Long-term outcome of combined radiologic and surgical strategy for the management of biliary complications after pediatric liver transplantation
Source: BMC Res Notes. 2024 Mar 20;17:86. doi: 10.1186/s13104-024-06735-6 (PMC10953252; doi:10.1186/s13104-024-06735-6)
Supplement: Supplementary file 5 — Additional file 5. Patient and liver graft survival within the pediatric liver transplantation cohort and biliary complications cohort. [file 13104_2024_6735_MOESM5_ESM.docx]

**Additional Material 5**

Patient and liver graft survival within the pediatric liver transplantation cohort and biliary complications cohort.

p=0.001

A. Patient survival

p=0.889

B. Graft survival
