# Supplementary material for: Long-term outcome of combined radiologic and surgical strategy for the management of biliary complications after pediatric liver transplantation
Source: BMC Res Notes. 2024 Mar 20;17:86. doi: 10.1186/s13104-024-06735-6 (PMC10953252; doi:10.1186/s13104-024-06735-6)
Supplement: Supplementary file 6 — Additional file 6. Treatment protocol for percutaneous transhepatic balloon cholangioplasty in pediatric liver transplantation and success rates. ERCP: endoscopic retrograde cholangiopancreatography, LT: liver transplantation, n.a.: not applicable. [file 13104_2024_6735_MOESM6_ESM.docx]

**Additional Material 6** Treatment protocol for percutaneous transhepatic balloon cholangioplasty in pediatric liver transplantation and success rates. ERCP: endoscopic retrograde cholangiopancreatography, LT: liver transplantation, n.a.: not applicable

| **Article** | **Nr. px** | **Treatment protocol** | **Number of procedures** | **Drain duration** | **Follow-up** | **Success** |
| --- | --- | --- | --- | --- | --- | --- |
| Anderson CD, 2010(1) | 17 | 16 Internal/External biliary drainage catheter, dilatation and drain change to maximal size  1 patient ERCP | 80% multiple interventions  30% >4 interventions | 6-9 months | 29.8 months (2-90) | 71%  29% failed percutaneous treatment (4 surgery and 1 Re LT) |
| Belenky A, 2004(46) | 7 | Internal/External biliary drainage catheter, dilatations every 10-20 days | 2-3 sessions | 1-2 months | 27 months (12-54) | 100% (clinical: resolution of stenosis, normal liver enzymes, no clinical symptoms) |
| Cardarelli-Leite L, 2017(22) | 7 | Internal/External biliary drainage catheter, procedure repeated 2-3 months after | 2 px – 2 dilatations  3 px – 3 dilatations  2 px – 2 dilatations | 5.8 months (range 3.1 – 12.6) | 15.4 months (5.3 – 26.7) | 85.7% technical success at 1^st^ attempt  100% (clinical) |
| Dulcetta L, 2022(16) | 78 | Internal/External biliary drainage catheter, 3 dilatations if needed every 3 months | n.a. | 31 days (IQR 54.4 days) | n.a. | 87.9% technical success at 1^st^ attempt  94.1% technical success at 2^nd^ attempt |
| Feier F, 2014(33) | 43 | Internal/External biliary drainage catheter, treatment sessions every 2 months  2 patients ERCP | 3.9±1.98 | 8 months (40 days – 38 months) | 39.5 months (2.2 months – 13.8 years) | 78% (≤30% residual stricture, biliary emptying time ≤3 min)  11% Re LT |
| Imamine R, 2015(35) | 52 | Internal/External biliary drainage catheter, dilatations every 1-2 weeks  ERCP | 1-4 sessions dilatations | 4 months (1-31) | 10 years | 83 % clinical success (no residual stenosis, free contrast passage); primary patency rates 75% -70%-70%-68% at 1-3-5-10 years |
| Jarzebicka D, 2017(36) | 67 | External biliary drainage catheter, dilatation every 3 months +/- 4 weeks  ERCP  Surgery | n.a. | n.a. | n.a. | 89% overall success (no residual stricture, free passage of contrast) |
| Karakayali F, 2013(47) | 26 | Internal/External biliary drainage catheter  Surgery | n.a. | n.a. | n.a. | 73% |
| Kling K, 2004(7) | 16 | Stent placement, repeated radiologic procedures  Surgery | 11.8±14.2 procedures (median 6.5, range 2-45) | 8.9±15.2 months (median 8.5, range 2-58) | 36±22 months | n.a. |
| Lee AY, 2020(18) | 42 | Endoscopic, dilatation every 4-6 weeks, stent placement  Internal/External biliary drainage catheter, dilatations every 4-6 weeks | 2-4 dilatations | 77-82 days | 13.8 years (1.2-30 years) | 60% success (technical), 20% surgical revision  20% re transplantation |
| Lorenz JM, 2001(37) | 76 | Internal/External biliary drainage catheter | n.a. | n.a. | n.a. | 100% if dilated  92% if non dilated (technical) |
| Marra P, 2022(28) | 6 | Percutaneous biodegradable stent | n.a. | n.a. | 271 days (IQR 120.5) | 100% technical success  1 stent dislodgement |
| Miraglia R, 2008(38) | 27 | Internal/External biliary drainage catheter, dilatations performed every 4 weeks, minimum 3 | 4 dilatations | n.a. | 15 months (2-46) | 75% (clinical) |
| Moreira AM, 2010(23) | 64 | Internal/External biliary drainage catheter, dilatations every 2 months, catheter removed after min 6 months | n.a. | 65.7% - 10 months drainage  20% - 24 months 11.4% - 44 months | 5.5 years (15 days-1595 days) | 82.6% (clinical) |
| Oggero AS, 2022(39) | 14 | Internal/External biliary drainage catheter every 15 days | 3 sessions | n.a. | 35.7 months ± 21.1 months | Succes rate 85.7%, 70% and 70% at 1, 3 and 5 years (no definition) |
| Prajapati HS, 2016(34) | 58 | Internal/External biliary drainage catheter, dilatations every 3-4 weeks | n.a. | 6 months | 48.4 months (0.57 – 145.6 months) | 55.6% |
| Reis SR, 2019(48) | 34 | Internal/External biliary drainage catheter  3 session dilatation protocol every 2 weeks vs multidisciplinary team | n.a. | 49 days vs 89 days | 5 years (1 day - 11.2 years) | 80% vs 61.1% success |
| Sunku B, 2006(27) | 35 | Dilatation, stent for minimum 3 months | n.a. | n.a. | 4.4 years(0.5-5.9) | 34% for the 1^st^;  40% for the 2^nd^ |
| Valentino P, 2020(17) | 113 | Radiologic 66%  ERCP 29%  Surgical 4% | n.a. | n.a. | 3.9 years | 92% stricture resolution (no intervention for 6 months) |
| Yan K, 2021(15) | 50 | Internal/External biliary drainage catheter, cholangiogram every 10-12 weeks. | 5 dilatations (2-41) | 152 days (76-308) | n.a. | 78% (technical success and laboratory) |
